# Aligning femtech innovation With equity: a strategic framework for real world impact

**DOI:** 10.3389/fgwh.2026.1762741

**Published:** 2026-05-18

**Authors:** Shirin Ashrafganjouei, Lindsay Eziashi, Muskaan Bhan, Roswitha Priscilla Verwer, Melissa Paola Mezzari

**Affiliations:** YON E Health Innovations B.V., Amsterdam, Netherlands

**Keywords:** AI in reproductive health, barriers in femtech, femtech innovation, inclusive health data, point-of-care testing, strategic framework for femtech, wearables and continuous monitoring, women's health equity

## Abstract

Femtech is advancing rapidly as a field with the capacity to transform women's health by addressing prevailing inequities in diagnosis, care, and access. Despite systemic, structural, cultural, data, regulatory, and access barriers that have historically limited progress, recent innovations are demonstrating measurable impact. Advances in point of care diagnostics and CRISPR based technologies promise earlier and more precise detection. Wearables and continuous monitoring are expanding opportunities for personalized care, while artificial intelligence, the Internet of Things, and real world data integration are creating new pathways for evidence generation, adaptive decision support, and population level insights. This article synthesizes these developments into a conceptual framework to guide future research, policy, and implementation in femtech, rather than aiming to provide original clinical data. It maps how femtech innovation is reshaping the landscape while highlighting areas where alignment with diverse user needs and infrastructures remains essential. To accelerate translation from innovation to execution, this article proposes a four pillar framework as a strategic roadmap: (1) building inclusive and representative evidence bases, (2) ensuring usability and affordability across contexts, (3) strengthening regulatory and ethical alignment, and (4) fostering integration of data and delivery systems. By situating technological advances within this framework, femtech can become a coherent, equitable, and scientifically robust ecosystem. This evolution holds the potential to close diagnostic and informational gaps and redefine global standards of care. Understanding and addressing these intersecting dimensions will guide research priorities, investment strategies, and policy development, ensuring that femtech fulfills its promise with rigor, inclusivity, and impact.

## 1 Introduction

Femtech has emerged as a transformative force in women's health by reframing prevailing inequities as innovative, solvable challenges instead of simply unavoidable outcomes. What began as a loosely defined sector has evolved into a multidimensional ecosystem extending across diagnostics, wearables, digital infrastructures, and AI enabled platforms (1, 2). These technologies share a unifying purpose: generating timely, individualized health information that strengthens prevention, early detection, and informed clinical decision making (3). Many achieve this by leveraging rapid and portable systems (i.e., CRISPR-based diagnostics) to analyze low concentration biomarkers including single-nucleotide polymorphisms (SNPs) and cell free DNA (cfDNA) (4, 5). Consequently, femtech does not represent a niche category, but an equity driven solution space that links access, education, funding, and ethics with technological innovation (4, 6). This is achieved primarily through the development of accessible point of care (POC) systems designed to meet the World Health Organization (WHO) ASSURED criteria, which ensure that POC tests are Affordable, Sensitive, Specific, User friendly, Rapid and Robust, Equipment free, and Deliverable to end users (4, 6). The need for such an ecosystem becomes clear against the backdrop of persistent gender inequities in health research and care delivery (7–9). Decades of underrepresentation in clinical trials, structural underfunding of female specific conditions, and gaps in sex disaggregated data have constrained the evidence base that guides diagnostics and treatment (10–12). Although regulatory frameworks have improved, these historical patterns continue to shape contemporary innovation pipelines and contribute to diagnostic delays, misclassification (such as diagnostic biases in pulse oximetry or standard vaginitis procedures), and inadequate personalization of care (4, 13). Here, we explore how femtech can help closing these gaps, while also identifying the structural and translational barriers that persist, such as challenges in meeting regulatory criteria and in achieving commercial success. The scope is conceptual and strategic, aimed at interpreting emerging advances across nucleic acid (NA)-based diagnostics, wearables, and AI-enabled tools through a barrier-informed lens. Rather than presenting original data, this work synthesizes contemporary evidence to clarify why technical maturity does not always translate into equitable impact across diverse populations. We focus on how systemic inequities (e.g., algorithmic bias), privacy concerns, lack of diversity in Real World Data (RWD), and regulatory uncertainties (especially for novel diagnostics and therapeutics) shape the adoption and effectiveness of new technologies. By synthesizing these themes, we outline a forward looking framework for guiding research priorities, strengthening evidence pipelines through robust analytical and clinical validation studies, and accelerating equitable translation. The goal is to articulate how femtech can evolve into a coherent, scalable, and globally relevant component of modern women's health, particularly by translating diagnostics into accessible point of care solutions for low-resource settings.

## 2 The barriers fueling femtech

Femtech's rapid growth is frequently celebrated as evidence of a long overdue shift toward addressing women's health needs. However, this momentum continues to be constrained by entrenched barriers currently recognized as systemic, structural, cultural, data related, regulatory, and access-based. Because of their existence, they continue to shape who benefits from innovation, which technologies receive investment, and how effective solutions reach diverse populations. Understanding these barriers is essential, not only to contextualize current limitations within the field, but also to inform the design of technologies that are equitable, clinically robust, and adaptable across global health systems.

1. Systemic barriers stem from persistent gender inequities in research and care, including exclusion of women from clinical studies, underfunding of diseases that disproportionately affect women, and limited representation of female specific physiology in innovation pipelines (14, 15). Additionally, women have been disproportionately affected by different types of diseases that remain underrepresented in biomedical research due to atypical presentation of symptoms (16). Underfunding and insufficient research directly constrain the development of diagnostics, therapeutics, and data driven tools tailored to women (14, 15).
2. Structural barriers reflect fragmented health systems, limited specialist services, inadequate reimbursement mechanisms, and poor digital infrastructure (16). Such disconnected systems make it difficult to scale femtech beyond affluent or well connected settings and risk reinforcing existing gender-based disparities if not addressed through investment in digital literacy, interoperability, and inclusive funding mechanisms (17, 18).
3. Cultural barriers center on stigma and silence around menstruation, sexual health, pelvic pain and menopause. Such barriers often hinder open discussions, reduce willingness to seek support, and limit adoption of technologies addressing intimate topics (19, 20). Cultural dynamics shape how patients and clinicians communicate, and they frequently influence how technologies are designed and marketed. As a result, even technically robust tools can fail if they do not align with users norms, comfort, and information needs (19).
4. Data barriers pose growing challenges in the era of AI enabled health technologies. Women particularly from low income, minority, or non Western populations remain underrepresented in health datasets, clinical registries, and digital health records (21). This leads to algorithmic bias, reduced diagnostic accuracy, and tools that do not generalize across skin tones, body types, and contexts (22, 23).
5. Regulatory barriers often arise from systems that are outdated or fragmented. These frameworks have not kept pace with hybrid wellness medical products, the handling of sensitive reproductive data, or the use of AI in decision support. As a result, questions remain about how such tools should be classified, whether they are safe, how privacy is protected, and how reimbursement is handled (24). At the same time, platform policies and content moderation practices can restrict dissemination of sexual and reproductive health information and products, limiting visibility and user education even when tools are evidence based and compliant (25, 26).
6. Access barriers highlight inequities at the intersection of technology and global health. These are most acute in low and middle income countries (LMICs) and among marginalized groups. Challenges with internet connectivity, affordability, language barriers, disability, and digital literacy often overlap, making it harder for women to benefit from wearables, diagnostic tools, and apps (24). These technologies can only deliver real benefit when they are adapted to local infrastructure and the social realities of the communities they aim to serve (18, 27, 28).

**Figure 1:**
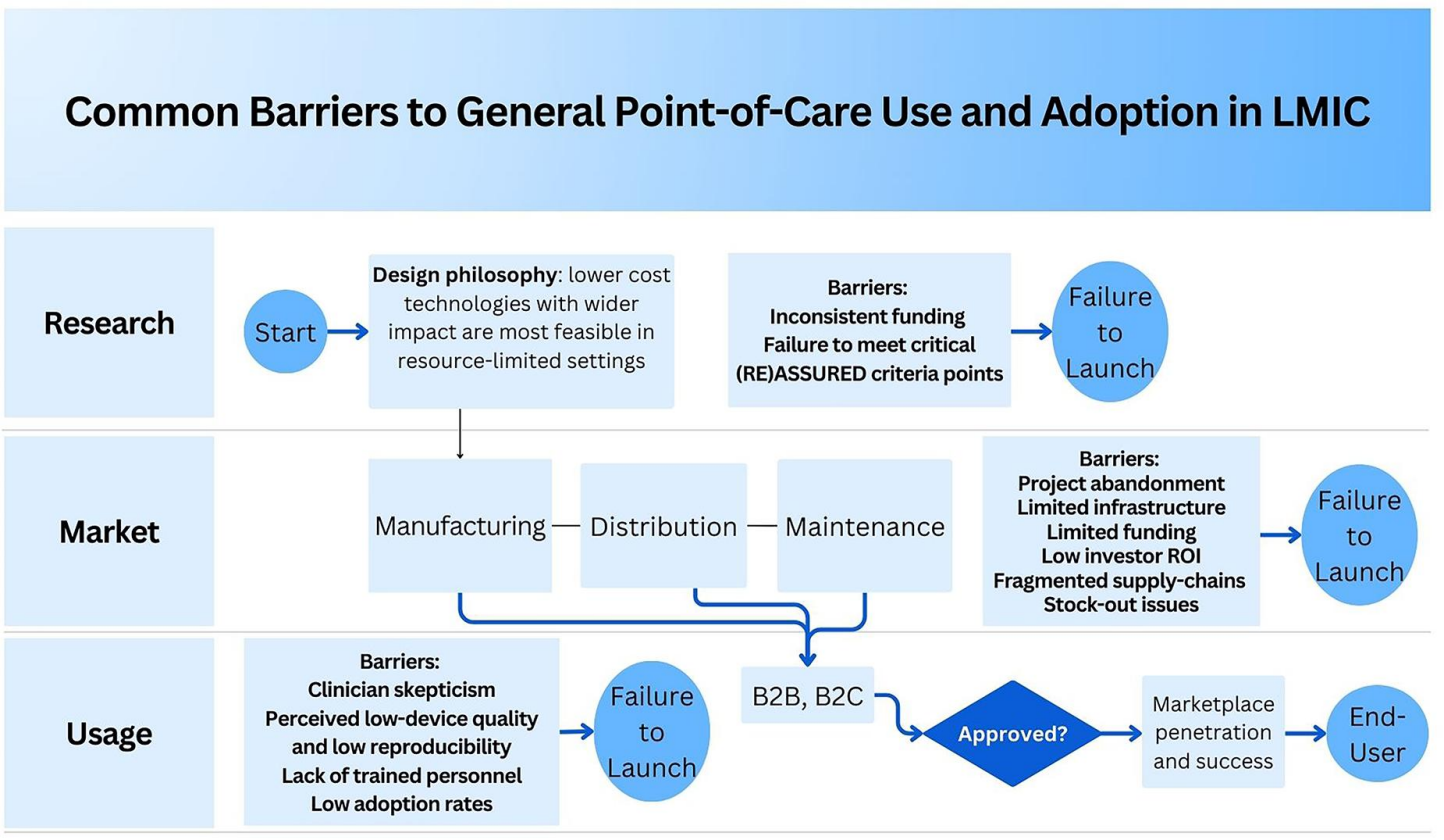
Common barriers affecting the development, distribution, and adoption of point-of-care (POC) diagnostic technologies in low- and middle-income countries (LMICs).

Together, these six barriers reveal that technological innovation alone is insufficient. Meaningful progress in femtech depends on simultaneous advances in research funding, inclusive datasets, culturally attuned design, strong privacy and regulatory safeguards, and context aware implementation strategies. Table 1 summarizes each barrier with concise descriptions, examples, potential implications, and corresponding solution pathways to keep the narrative streamlined while preserving concrete detail. A visual summary of these multi level barriers is shown in Figure 1, which maps how systemic, structural, cultural, data, regulatory and access constraints interact across the femtech lifecycle: from research and product development through market entry and everyday use in LMIC settings.

**Table 1:** Summary of systemic, structural, cultural, data, regulatory, and market barriers affecting femtech innovation and adoption. Each barrier type is illustrated with descriptions and examples, highlighting implications for equity, trust, and scalability, alongside proposed solution pathways and supporting references.

Barrier Type	Description	Examples (References)	Possible Implications	Solutions
Systemic	Persistent gender inequities in health research, funding, and innovation pipelines leading to biased prioritization and exclusion of women's health needs.	Underfunding of endometriosis, migraine, PMS, menopause (15); exclusion of women in clinical trials (14); female physiology underrepresented in innovation (12, 48).	Limited evidence base; slow innovation; narrow femtech application scope; reduced accuracy and trust in AI and diagnostics.	Increase research funding for under-recognized diseases; mandate sex-and-gender inclusive research and development; diversify AI teams and engagement.
Structural	Fragmented health systems, poor digital infrastructure, and inadequate reimbursement impair femtech scaling and service integration.	Limited specialist services in rural/LMICs (16); lack of data interoperability across clinical systems (18); insurance exclusions (17).	Concentration of femtech tools in affluent settings; barriers to access and sustained use.	Invest in interoperable infrastructure and funding pathways; improve digital literacy and point-of-care integrations.
Cultural	Stigma and taboos around menstruation, sexual health, and menopause block discourse and technology adoption.	Shame around menstruation and pelvic pain (20); discomfort with vaginal devices (19); communication breakdowns between patients and clinicians (20).	Decreased help-seeking; low product uptake; misinformation persists.	Community-engaged co-design; menstrual health education; stigma-sensitive clinician training.
Data	Underrepresentation in datasets and limited quality data lead to algorithmic bias and poor predictive validity.	Lack of menstrual and reproductive data standards (21); bias in skin lesion AI classification (23); single-center validation studies (22).	Algorithmic bias; inaccurate diagnostics for diverse groups; erosion of trust.	Expand inclusive datasets; multi-site validation; fairness-aware AI models.
Regulatory	Ambiguous or outdated oversight for hybrid digital-wellness and reproductive technologies.	Unclear classifications (24); privacy risks e.g., reproductive data misuse (26); limited clinical validation guidance (24).	Slow commercialization; user mistrust; uneven safety and quality controls.	Clarify regulatory frameworks; enforce quality control for AI; embed privacy by design.
Access/Market	Geographic, economic, linguistic, and digital divides hinder adoption and equity.	Infrastructure and skills gaps in LMICs (27); affordability barriers (24); racism and language bias in AI tools (28); content moderation restricting sexual health ads (25, 26).	Inequitable uptake; femtech focused on urban, English-speaking, wealthier populations.	Low-cost, offline-capable tools; culturally attuned interfaces; policy advocacy for fair content policies.

## 3 Mapping advances in femtech innovation

Femtech innovations are advancing rapidly across diagnostics, wearable biosensing, and AI driven data systems, offering new pathways to reduce long standing inequities in women's health. Yet scientific progress does not automatically translate into real world impact. Each technological advance must be understood within the constraints outlined as barriers in Section 2, which continue to shape how and where innovation is adopted. This section synthesizes the most recent developments and highlights where progress is strongest, where evidence is emerging, and where translation remains limited. It applies a three-tier maturity framework: Consistent Progress, Emerging Evidence, or Limited Translation; linking technical advances to six systemic barriers that may shape their trajectory. It draws on a targeted narrative review of point-of-care diagnostics, CRISPR, wearable biosensors, and AI tools, prioritizing peer-reviewed studies with clinical validation. This approach highlights where structural “leaks in the pipeline” continue to hinder equitable translation into real-world health impact. The following sections highlight where progress is most consistent, where evidence is emerging, and where translation into practice remains limited.

### 3.1 Point of care diagnostics and CRISPR based innovation

Point of care (POC) diagnostics are shifting women's health screening away from centralized laboratories toward decentralized clinical and home based environments. CRISPR based platforms have been central to this transition. Cas12/13 driven assays embedded in microfluidic cartridges now achieve highly sensitive nucleic acid detection with minimal sample preparation, enabling rapid screening for high risk HPV and other pathogens at the point of care (29–31). Multiplexed CRISPR-RDB systems reach performance levels approaching PCR, yet remain portable and cost effective attributes particularly relevant for low resource settings. The validity of the progress in CRISPR-based assays is supported by their high sensitivity and portability. However, when interpreted through the lens of Access Barriers, their clinical utility remains ‘Consistent’ only for centralized settings, while remaining ‘Emerging’ for low-resource settings due to the lack of multi-site validation in LMIC infrastructures.

Menstrual effluent represents a parallel and rapidly growing diagnostic modality. Rich in lipidomic and proteomic biomarkers, menstrual fluid supports non-invasive screening for gynecologic conditions such as endometriosis. Recent studies report sensitivities of 80%–85% for lipidomic signatures distinguishing affected individuals from controls (32). Complementary approaches, such as borophene nanosheet biosensors detecting HMGB1 with increased sensitivity, further demonstrate how menstrual blood can facilitate decentralized testing and longitudinal disease monitoring (33).

AI enhanced imaging is also redefining diagnostic capacity. Tools such as Mia (Mammography Intelligent Assessment) integrate machine learning models into mammographic workflows to reduce false-negative rates and support earlier detection, including in dense breast tissue (34). Preliminary robotic ultrasound systems, such as those developed by EndoCure, have demonstrated sub millimetre lesion detection for endometriosis, suggesting a route to less invasive, image guided diagnosis (35). Equitable deployment of these systems will require validation across diverse racial and ethnic populations and across clinical settings with varying levels of expertise. While AI-driven models for symptom clustering demonstrate high technical accuracy, our interpretation suggests ‘Limited Translation’ validity because these models often rely on demographically homogeneous datasets, potentially reproducing algorithmic bias when applied to diverse skin tones or body types.

Across these diagnostic innovations, three themes consistently emerge: decentralization, reduced reliance on laboratory infrastructure, and improved accessibility. The challenge ahead lies in ensuring rigorous multi-site validation and adaptation to varied cultural, clinical, and infrastructural contexts.

### 3.2 Wearables and continuous monitoring

Wearable technologies have expanded from consumer oriented fitness trackers into multimodal biosensing platforms capable of capturing biochemical, hormonal, immunologic, and cardiovascular signals in real time. These systems integrate anomaly detection and predictive analytics, enabling longitudinal monitoring far beyond fertility tracking (36).

Innovation in device form factors is accelerating this transition. Microneedle patches containing lyophilized CRISPR reagents can passively capture viral DNA or RNA from environmental exposure, supporting decentralized infectious disease surveillance (37). Similarly, nanotechnology based hormone assays now achieve laboratory level sensitivity in at home settings, providing continuous endocrine monitoring at unprecedented resolution (36).Intravaginal biosensors mark another major step toward precise reproductive health monitoring. They can measure vaginal pH and basal body temperature, metrics shown to vary across ethnic groups and to influence fertility tracking accuracy (38). Bioresorbable pH sensing rings support *in situ* monitoring of vaginal health and transmit data wirelessly, addressing previous concerns around hygiene, device removal, and comfort (39). Meanwhile, flexible microfluidic and graphene-based devices enable ongoing measurement of electrolytes, metabolites, and hormonal biomarkers, though large scale usability and validation studies remain pending (36).

### 3.3 AI, IoT, and real world data integration

Transformative femtech advances integrate heterogeneous data streams, such as biomarkers, wearables, and electronic health records (EHRs), into analytics that anticipate health trajectories and guide clinical decisions.EHR based predictive models show promise by identifying endometriosis symptom clusters years before diagnosis, potentially reducing the current 7–11 year diagnostic delay (40). Large language model (LLM) systems extend this by accurately extracting symptom descriptors from unstructured notes for digital phenotyping (41). However, these tools ofter rely on narrow, demographically homogenous datasets and lack prospective validation, raising concerns about generalizability and fairness (21). Without mitigation, algorithmic decisions risk reproducing structural inequities already embedded in clinical workflows.

IoT frameworks support adaptive feedback loops and personalized interventions via continuous sensor synchronization. These systems require robust governance and interoperable architectures to prevent biased recommendations. Ultimately, AI-enabled femtech demonstrates strong technical maturity but remains limited by data inequity, fragmented infrastructure, and regulatory ambiguity. Ensuring equitable integration will require coordinated strategies that combine technical innovation with ethical, regulatory, and clinical oversight.

## 4 Building inclusive femTech through four pillars

The advances outlined in Sections 2 and 3 illustrate a striking tension at the centre of femtech: while scientific innovation in diagnostics, continuous monitoring, and AI enabled analytics is accelerating, translation into equitable clinical impact remains deeply uneven (16, 24). Systemic under representation, fragmented infrastructure, cultural stigma, data inequity, regulatory ambiguity, and limited access continue to dictate who benefits, when, and under what conditions (15, 21). Addressing these misalignments requires not only better technologies but deliberate structural reforms. In response, we propose a four pillar roadmap to guide a more coherent, trustworthy, and globally relevant femtech ecosystem.

### 4.1 Pillar 1: mandate inclusive, multi-site validation and bias aware regulation

Ensuring equity in femtech requires expanding the definition of validation beyond traditional performance metrics. All femtech modalities, including POC diagnostics, intravaginal biosensors, wearables, and AI based systems, must demonstrate reproducible performance across diverse clinical sites, racial and ethnic groups, age ranges, and socioeconomic contexts. Current evidence pipelines remain concentrated in high resource settings, resulting in limited insight into how technologies behave in LMIC infrastructures, in perimenopausal populations, or among users with low digital literacy. To transition from conceptual frameworks to clinical reality, Femtech must adopt mandatory bias audits, pre-market multi-site requirements and post-deployment monitoring for adaptive AI tools that encompass the racial, ethnic, and socioeconomic diversity identified in current literature. Pragmatic trial designs, LMIC based validation hubs, and sex disaggregated reporting standards are essential to strengthen both scientific credibility and public trust.

### 4.2 Pillar 2: build ethical, interoperable, and privacy preserving data infrastructure

Interoperability challenges, fragmented data governance, and opaque data sharing practices undermine the integration of femtech tools into clinical workflows. Health Level Seven Fast Healthcare Interoperability Resources (42) provides a standards based framework of APIs and data models intended to support secure exchange of clinical information across systems (42, 43). Although HL7 FHIR standards and mandates under the 21st Century Cures Act provide a technical foundation for connectivity, reproductive health datasets remain siloed and inconsistently structured. At the same time, empirical audits of menstrual and reproductive health applications show that a substantial proportion share sensitive user data with analytics and advertising third parties, often without meaningful, informed consent (26, 44), posing risks for discrimination and erosion of trust.

We state that an equitable femtech ecosystem requires three structural reforms:

1. Technical interoperability through universal FHIR adoption and standardized reproductive health Common Data Elements.
2. Trustworthy governance via General Data Protection Regulation (GDPR) aligned consent models, data trusts, and cooperative stewardship frameworks that return meaningful control to women.
3. Privacy-preserving analytics, including federated learning and differential privacy, to enable collaborative research without compromising user safety.

Together, these components form the backbone of a secure, user centered data environment capable of supporting high-quality evidence generation and cross platform integration.

### 4.3 Pillar 3: embed human centered, culturally responsive Co-design

Technical capability alone cannot guarantee adoption, adherence, or health impact. Femtech solutions must be designed in close partnership with the women who will use them, reflecting varied cultural norms, linguistic needs, comfort levels, disability requirements, and reproductive histories. Evidence from tools such as My Healthy Pregnancy and My Decision shows that participatory design steering committees with users, iterative testing with diverse demographic groups, and explicit attention to historical injustices substantially improve engagement, trust, and clinical relevance (45).We propose that femtech developers systematize co-design by incorporating user panels, community partnerships in LMIC settings, and evaluation frameworks that explicitly measure cultural acceptability, informational equity, and psychological safety. A generic design approach is incompatible with the complexity and diversity of women's lived experiences.

### 4.4 Pillar 4: foster equitable funding mechanisms and strategic partnerships

As described in Section 2, persistent underinvestment in women's technologies and male-dominated investment networks constrain femtech's clinical trajectory. Less than 0.5% of healthcare venture capital is allocated to women's health (15, 46), limiting the advancement of technologies that address high burden conditions such as endometriosis, menopause, and pelvic pain (12, 14). We argue for coalition-based financing models that unite public funders, private investors, philanthropic organizations, and global health agencies (47). Public investment should de-risk early regulatory and clinical validation; philanthropic partners can prioritize inclusion focused pilots; and private capital can accelerate commercialization once feasibility is established (24). Strategic partnerships with hospital networks, academic institutions, LMIC health ministries, and NGOs are essential for accessing representative datasets, enabling decentralized clinical trials, and developing culturally grounded deployment strategies (16, 28).

## 5 Conclusion

Despite impressive technological progress, several unresolved gaps continue to limit femtech's translation into equitable clinical impact. Evidence pipelines remain narrow: most diagnostics, wearables, and AI enabled tools lack prospective, multi site validation, particularly among populations historically excluded from women's health research. This limitation is further underscored by the article's conceptual nature and reliance on secondary synthesis rather than primary clinical data. Cultural acceptability, long-term usability, and safety of emerging intravaginal and continuous monitoring devices also require far deeper investigation into historical systemic underfunding and socio-cultural taboos. These gaps point to a field where innovation has advanced faster than regulatory, ethical, and implementation science can support, thus representing a temporal snapshot of a field in which technical thresholds and regulatory landscapes continue to evolve. Addressing these limitations defines the next phase of femtech research, which requires transitioning from conceptual models toward empirical validation. Priority directives include developing harmonized reproductive health data standards; conducting large, demographically diverse validation studies; establishing regulatory science frameworks for adaptive AI systems; and investing in culturally grounded user experience research. Equally essential is designing implementation models suited for low-resource settings, where infrastructural constraints shape feasibility more than technical performance. While the proposed framework emphasizes the importance of multi-site and demographically diverse validation, its broader applicability will require further prospective evaluation across varied clinical and geographical infrastructures to confirm its universal validity.

## Data Availability

The original contributions presented in the study are included in the article/Supplementary Material, further inquiries can be directed to the corresponding author.
